# Crystal structure and interactions of the Tof1–Csm3 (Timeless–Tipin) fork protection complex

**DOI:** 10.1093/nar/gkaa456

**Published:** 2020-05-29

**Authors:** Daniel B Grabarczyk

**Affiliations:** Rudolf Virchow Center for Experimental Biomedicine, Institute for Structural Biology, University of Würzburg, Josef-Schneider-Str. 2, Würzburg, Germany

## Abstract

The Tof1–Csm3 fork protection complex has a central role in the replisome—it promotes the progression of DNA replication forks and protects them when they stall, while also enabling cohesion establishment and checkpoint responses. Here, I present the crystal structure of the Tof1–Csm3 complex from *Chaetomium thermophilum* at 3.1 Å resolution. The structure reveals that both proteins together form an extended alpha helical repeat structure, which suggests a mechanical or scaffolding role for the complex. Expanding on this idea, I characterize a DNA interacting region and a cancer-associated Mrc1 binding site. This study provides the molecular basis for understanding the functions of the Tof1–Csm3 complex, its human orthologue the Timeless–Tipin complex and additionally the Drosophila circadian rhythm protein Timeless.

## INTRODUCTION

As well as error-free DNA synthesis, the eukaryotic replication fork must coordinate processes such as establishing chromosome cohesion, activating the S phase checkpoint and transferring epigenetic material. Furthermore, the polymerases and helicases which form the core of the replication machinery are regulated to couple unwinding and synthesis, protect the fork when it is blocked and integrate external signals. The coordination of all of these processes is required to maintain genome stability (1). This becomes especially important in conditions of replication stress, which is a common feature of cancer cells (2,3). Indeed many non-essential replisome-associated proteins are upregulated and become essential in cancer (4–6).

The Tof1–Csm3 fork protection complex was identified as a non-essential chromosome cohesion factor (7,8) that interacts with Topoisomerase 1 (9) to link concatenation and fork regulation (10). Additionally it has an important role in protecting stalled forks and enabling their restart (11–13), coupling the replicative helicases and polymerases (14), promoting fork progression (15,16), mediating the S phase checkpoint (17,18) and maintaining genome stability at CAG repeats (19). The mammalian orthologue of Tof1–Csm3 is the Timeless–Tipin complex (20), which has similar functions (21–24) and additionally regulates the fork in response to oxidative stress (25). Timeless also has a PARP1 binding (PAB) domain at its C-terminus important for double-stranded break repair (26,27), and interacts with RPA through the C-terminus of Tipin (28,29). Timeless was first discovered as a circadian rhythm regulator in drosophila (30). However, drosophila also contain a homologue of mammalian Timeless, known as Tim2, which is likely to be the true orthologue of Tof1 (31). Nevertheless, it has been suggested that mammalian Timeless links the circadian rhythm with DNA replication (32,33).

A lack of structural information has precluded progress in the understanding of the specific role that Tof1–Csm3 plays at replication forks, and how this relates to the diverse phenotypes resulting from its mutation. A crystal structure of a the N-terminal domain of Timeless shows that this forms an Armadillo repeat protein (34), while 2D cryo-EM classes of the yeast replisome show that Tof1–Csm3 binds in front of the fork to stabilize incoming DNA (35). Here, I present the structure of the *Chaetomium thermophilum* Tof1–Csm3 complex. The structure reveals that the protein is folded as a single unit, with Csm3 forming an alpha-helical bundle that caps the Armadillo repeats of Tof1. This suggests a structural role for this complex at the fork. The crystallographic packing in my structure reveals a peptide-binding cleft that is affected by a cancer-associated mutation in human Timeless. I show this cleft interacts with a section of Mrc1. Furthermore, I map a double-stranded DNA binding activity to a concave basic patch at the Tof1–Csm3 boundary. The structure also enables sequence alignment of Tof1 with human and drosophila Timeless clarifying the similarity of their structures but differences in function.

## MATERIALS AND METHODS

### Molecular biology

The entire *C. thermophilum TOF1* and *CSM3* genes were synthesized by ATG:biosynthetics and provided in separate pGH vectors. For all expression constructs a pETM-14 vector (EMBL) with an N-terminal His6-tag was used. *TOF1* and *CSM3* were cloned into the multi-cloning site for co-transcription and Csm3 was untagged but cloned in with a ribosomal binding sequence. Construct design was guided by disorder prediction by the DISOPRED server (36). All primers (Sigma-Aldrich) are listed in Supplementary Table S1. First Tof1(1–728) was amplified with primers P1 and P2, and inserted by restriction cloning into pETM14 using NcoI and BamH1 to generate pETM-14-Tof1. Csm3(48–157) or Csm3(77–157) was then amplified with primers P3 and either P4 or P5 to generate either pETM14–Tof1–Csm3 or pETM14–Tof1–Csm3trunc. Loops were deleted from Tof1 by blunt-end mutagenesis. For DL1, residues 256–363 were replaced by one glycine using primers P6 and P7. For DL1a, a construct containing DL1 was further mutated using primers P8 and P9 resulting in replacement of residues 256–259 by a single glycine. For DL2, residues 420–434 were removed with primers P10 and P11. For DL3, residues 558–585 were replaced by one glycine using primers P12 and P13. The C-terminal domain of Tof1 (residues 728–900) was cloned into pETM14 with restriction enzymes BamHI and EcoRI using primers P14 and P15. The same approach was used to C-terminally elongate Tof1–Csm3. In this case, the stop codon and BamHI site were then removed by blunt-end mutagenesis using primers P16 and P17 to generate pETM14–Tof1long–Csm3. For DL4, residues 784–813 were replaced by a single glycine using primers P18 and P19. To generate Tof1–Csm3 constructs where residues 10-612 of Tof1 were removed, blunt-end mutagenesis was performed with primers P20 and P21 using either pETM14–Tof1–Csm3 or pETM14-Tof1long-Csm3 as templates to generate pETM14-NtruncTof1–Csm3 and pETM14–NtruncTof1long–Csm3. The quadruple alanine substitutions were generated by sequential blunt-end mutagenesis using primers: K50A—P22 and P23, R54A—P24 and P25, R98A—P26 and P27, R173A—P28 and P29, R519A/K522A—P30 and P31, K697A/R698A— P32 and P33, Csm3–K94A/K96A—P34 and P35, Csm3–K132A/K136A—P36 and P37.

The three Mrc1 constructs were amplified from S. cerevisiae genomic DNA using primers: 1–400 – P38 and P39; 401–800 – P40 and P41; 801–1096 – P42 and P43. and then inserted into pETM-41 (EMBL) after N-terminal His6-tagged maltose binding protein.

### Expression and purification of Tof1–Csm3 and Mrc1 constructs

All Tof1–Csm3 and Mrc1 constructs were expressed and purified using the same method. The appropriate plasmid was transformed into BL21(DE3)Star (Novagen) cells and grown in media supplemented with 50 μg/ml kanamycin. Large terrific broth expression cultures were inoculated 1 in 100 with an overnight start culture and grown at 30°C with shaking at 200 rpm until they reached an *A*_600_ of 0.5. The temperature was reduced to 17°C, and cultures were induced with 0.4 mM IPTG overnight, followed by harvesting using centrifugation, and storage of bacterial pellets at –80°C until use. Thawed pellets were resuspended in 50 mM Tris–HCl pH 8.0, 500 mM NaCl, 1 mM TCEP, 10 mM imidazole, 1 EDTA-free protease inhibitor tablet/50 ml (Roche), and 30 U/mL DNase I. Lysis was performed by two passages through a Microfluidics M-110P microfluidizer at 150 MPa. The lysate was cleared by centrifugation for 1 hour at 60 000 × g and then loaded on a 5 ml Histrap FF column (GE Healthcare) equilibrated in 50 mM Tris–HCl pH 8.0, 500 mM NaCl, 1 mM TCEP, using an Akta Purifier FPLC system. The column was then washed with 16 column volumes of 25 mM imidazole in the same buffer, and then eluted with a 10 column volume gradient of 25–250 mM imidazole. Protein-containing fractions were then concentrated by microfiltration before loading on a Superdex 200 26/60 prepgrade column (GE Healthcare) column that had been equilibrated in 20 mM HEPES pH 7.5, 1 mM TCEP with different concentrations of NaCl for the different constructs as indicated in Supplementary Figure S1A (HBS-X). Fractions containing the desired protein were then concentrated using a spin concentrator and stored at -80°C. The protein concentration was estimated from the *A*_280_ absorption using the extinction coefficient calculated by the Expasy Protparam server (37).

### Crystallization

Crystallization trials were performed with a Honeybee fluid transfer robot using the sitting drop vapor-diffusion method with 0.3 μL of protein was mixed at a 1:1 ratio with mother liquor from customized screens (Supplementary Figure S1B). Drops were incubated at 20°C and equilibrated against 40 μl mother liquor supplemented with an additional 0, 125, 187 or 250 mM NaCl. The Tof1–Csm3 complex was crystallized at 30 mg/ml in HBS-500 and mixed with a precipitant solution containing 0.2 M Potassium Acetate, 4–8% PEG 20 000, 0.1 M Tris–HCl pH 7.5–8.5. The reservoir was supplemented with 125 mM NaCl. Crystals were cryo-protected in the same solution with 25% ethylene glycol additional.

### Data collection, structure solution and refinement

The data were collected at 100 K and at a wavelength of 0.980 Å at beamline P14 of the EMBL-operated PETRA III ring at DESY. The data were integrated using XDS (38), and then merged either using Aimless (39) to 3.6 Å or using the STARANISO server (40) to 3.09 Å (Table 1).

**Table 1.** Crystallographic data collection and refinement statistics

	Tof1–Csm3 STARANISO	Tof1–Csm3 spherical
Space group	C2	C2
**Cell dimensions**		
*a*, *b*, *c* (Å)	264.47, 82.03, 161.17	264.47, 82.03, 161.17
α, β, γ (^o^)	90.0, 124.85, 90.0	90.0, 124.85, 90.0
Resolution (Å)	76.74–3.09 (3.52–3.09)	80.50–3.60 (3.78–3.60)
R_pim_	0.078 (0.737)	0.093 (1.06)
CC_1/2_	0.996 (0.365)	0.997 (0.443)
I/Iσ	7.1 (1.2)	5.7 (0.9)
Completeness (%)	89.8 (46.3)^a^	99.3 (99.8)
Redundancy	7.0 (7.0)	7.0 (7.0)
**Refinement**		
Resolution	51.03–3.09	
No. reflections (free)	27151 (1353)	
*R*_work_/*R*_free_ (%)	23.7/26.4	
**No. atoms**		
Protein	15733	
Ligand/ion	0	
Water	0	
***B*-factors**		
Protein	123.4	
**r.m.s. deviations**		
Bond lengths (Å)	0.0081	
Angles (^o^)	0.920	
Molprobity Clashscore	2.80	
Ramachandran outliers (%)	0.21	
Ramachandran allowed (%)	95.9	

^a^Ellipsoidal completeness.

A homology model of *C. thermophilum* Tof1 residues 1-488 was generated from the structure of the corresponding region from human timeless (34) (PDB 5MQI) using the SWISS-MODEL server (41). From this, all sidechains were truncated to alanine and all loops deleted. Three copies were found using the STARANISO-processed data and PHASER (42) with a TFZ score of 11.1. Initial refinement was performed using Phenix Refine (43) and the non-corrected data to 3.6 Å. Helices were manually placed in the density, and Phenix Autobuild (44) was run occasionally and reduced model bias. A continuation of the alpha-helical repeat structure from the Timeless fragment was clear, and so this was exploited for sequence and topology assignment. The sequence was too short for the last four helices and so these were assigned to Csm3. Once the model was largely complete, refinement was continued using Buster (45,46) and the anisotropically corrected data to 3.1 Å. Intermittent cycles of molecular dynamic force-field refinement using the Namdinator server (47) proved essential for overcoming model bias, and the RaptorX evolutionary contact server (48) was used to validate the sequence assignment. The final *R*_work_/*R*_free_ values were 23.7/26.4% with 0.21% Ramachandran outliers, a Clashscore of 2.80 and an overall MolProbity score of 1.84 (49). The Chain A–Chain B Tof1–Csm3 copy was the best defined and used for all structural figures, unless otherwise stated, and these were generated using PyMOL (50).

### Electrophoretic mobility shift assays and native PAGE

The ssDNA oligonucleotide had the following sequence: 5′-Cy3-GTAGTTTGTACTGGTGACGA. The dsDNA substrate was generated by mixing this 1:1 with the complementary oligonucleotide 5′-TCGTCACCAGTACAAACTAC, melting at 95°C for 2 min, and then slow cooling at room temperature to anneal. DNA substrates were used at a final concentration of 50 nM. Samples were prepared in HBS-200 with an additional 10% glycerol, and incubated on ice for 30 min. Samples were loaded on a 6% polyacrylamide gel and run at 70 V for 50 min in a tris-glycine buffer system. After running, gels were scanned with a Pharos FX fluorescence imaging system (Biorad) and excitation/emission wavelengths of 532/605 nm. Native PAGE was performed with exactly the same buffers and method, except that gels were run for 2 h and then Coomassie stained.

## RESULTS

### Structure of the Tof1–Csm3 complex

To determine the structure of the Tof1–Csm3 complex, multiple constructs from the mildly thermophilic fungus *Chaetomium thermophilum* were screened for purification and expression. Crystals were obtained for many of these (Supplementary Figure S1A), but only formed in customized crystallization screens designed for challenging complexes. These screens are detailed in Supplementary Figure S1B, and were also previously used to crystallize another complex (51). The only construct of the complex resulting in diffracting crystals was Tof1(1–728)ΔL1,2,3–Csm3(48–157). Here, three predicted disordered loops were deleted from Tof1: L1 (256–363), L2 (420–434), L3 (558–585) (Supplementary Figure S1A). These crystals diffracted anisotropically to 3.1 Å (Table 1), and the dataset could be solved by molecular replacement using the N-terminal fragment of Timeless (PDB 5MQI) (34) with the remaining half of the protein manually built, exploiting molecular dynamics force-field refinement (47) and contact prediction (48). A final *R*_work_/*R*_free_ of 23.7/26.4% was achieved. The entire structure is well resolved (Supplementary Figure S2), aside from the N-terminal portion of Csm3, and two small loops in Tof1. The main crystal contacts occur at the N-terminus of Tof1, and thus Csm3 and the very C-terminus of Tof1 have relatively high B factors and there is lower map quality in this region (Supplementary Figure S2C).

**Figure 1.**
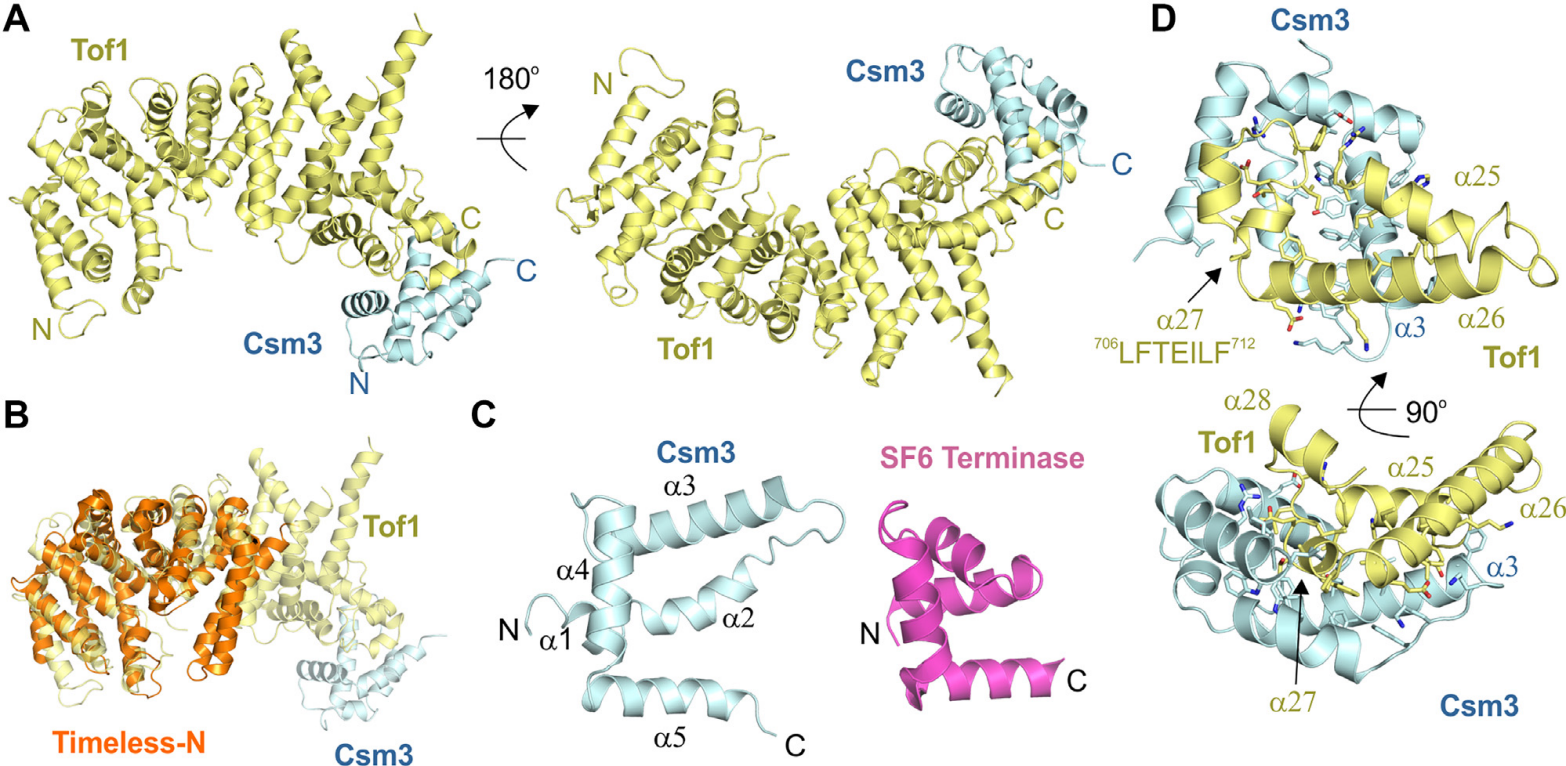
Crystal structure of the Tof1–Csm3 complex. (**A**) The complex is shown from two views with Tof1 colored in yellow and Csm3 in cyan. (**B**) Superposition of an N-terminal fragment of Timeless (PDB 5MQI) onto Tof1. (**C**) The structure of Csm3 and comparison to a protein with a similar fold, the DNA binding domain of SF6 small terminase (PDB 4ZC3). (**D**) Details of the interaction between Tof1 and Csm3. Only α25-27 of Tof1 are shown, and interfacial residues are shown in stick representation.

The structure reveals that, instead of containing distinct domains, the entire complex forms an extended alpha-helical repeat protein (Figure 1A). Tof1 begins with two helices linked by a beta-hairpin, which is then followed by eight three-helix armadillo repeats (Arm1–8). The previous Timeless N-terminal domain structure is a fragment of this structure ending after Arm-5. For this fragment, Tof1 and Timeless are clearly highly structurally related (Figure 1B). Csm3 further extends the alpha-helical repeat structure by forming a five-helix bundle which packs on the C-terminus of Tof1. Csm3 shows some similarity to a tetra-helical bundle helix-turn-helix fold, but the first helix is broken into two (Figure 1C). The closest structural homologue identified by PDBeFold (52) is the DNA binding domain of the small terminase from bacteriophage SF6 (Figure 1C) (53). However, in contrast to this domain, the helical bundle of Csm3 is flattened such that it no longer has a hydrophobic core and thus does not appear to be an independently folded structure and rather acts as a cap on the C-terminus of Tof1 (Figure 1C). The interface between the two is largely hydrophobic with some salt bridges, and consists of a large percentage of Csm3 (Figure 1D), showing why both proteins are required to stabilize each other (54). α25 and α26 from Arm-8 plus the following helix α27 contain all the interaction sites for Csm3 (Figure 1D). Tof1 helices α25 and α26 pack predominantly against Csm3 helix α3. Tof1 helix α27 inserts into the concave structure of Csm3 making hydrophobic interactions with Csm3 helices α2 and α3. The interface does not involve any of the residues previously predicted by a cross-linking/mass spectrometry analysis (34). These cross-linked residues lie predominantly in predicted disordered regions of the complex, which may have increased their cross-linking propensity.

### Relation of Tof1 homologues

**Figure 2.**
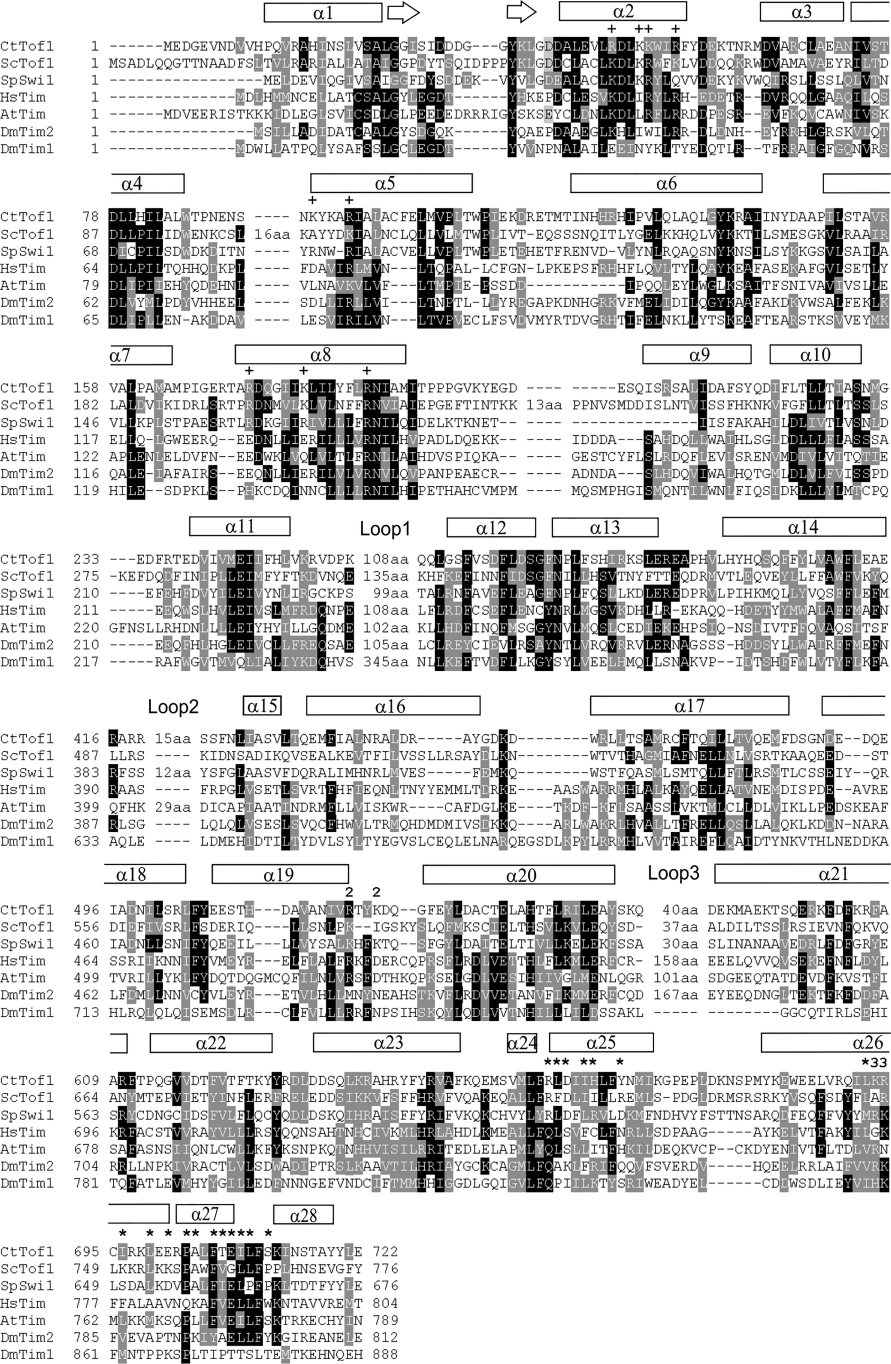
Sequence alignment of Tof1 with Timeless proteins. The alignment was performed using Clustal X2 (62) and displayed using BoxShade (63). Ct – *Chaetomium thermophilum*, Sc – *Saccharomyces cerevisiae*, Sp – *Schizosaccharomyces pombe* Hs – *Homo sapiens*, At – *Arabidopsis thaliana*, Dm – *Drosophila melanogaster*. Loop numbering refers to Supplementary Figure S1A. **+** Peptide interacting, * Csm3 interacting, **2** – BP2 mutations, **3 –** BP3 mutations.

Due to large variably sized loops and low sequence identity, it is challenging to unambiguously align the sequences of the various Tof1 homologues. With the structure, the alignment is clearer, and it is possible to gain structural and functional insight into the orthologous protein Timeless, and its homologue, the circadian rhythm regulator drosophila Timeless (CR-Timeless) (Figure 2). From this analysis it is immediately clear that the Timeless protein from varying eukaryotes has the same overall fold, with the hydrophobicity profile of all helices up to α26 conserved. Interestingly, helix α27, the major Csm3/Tipin interaction site, is very highly conserved in the DNA replication Tof1/Timeless proteins but absent in the drosophila CR-Timeless. This is clearly indicative of the separate function of this protein. Furthermore, Loop 1 is highly conserved in the DNA replication Tof1/Timeless proteins (Supplementary Figure S3B), but has an entirely different sequence in CR-Timeless. This suggests Loop 1 has an important role in DNA replication. The entire region of Csm3 in the crystallized construct shows high sequence conservation (Supplementary Figure S3A).

Interestingly, the presence of an additional small C-terminal portion of Tof1 (residues 728–900) largely increases the solubility of the Tof1–Csm3 complex, despite preventing crystal diffraction (Supplementary Figure S1). Thermofluor analysis of the Tof1(1–900)ΔL1,3–Csm3 construct reveals two denaturation transitions (Supplementary Figure S4A), suggesting it contains two independently folded domains. The Swiss-model server (41) identifies the C-terminal domain as the human Timeless PARP1-binding PAB domain (Supplementary Figure S3B). This domain can also be found in the CR-Timeless sequence (Supplementary Figure S3C), but not *Arabidopsis* Timeless. Confirming this assignment, the RaptorX server (48) which determines the fold *de novo* by evolutionarily predicted sequence-contact restraints, independently generates a structure from the *C. thermophilum* sequence which is highly similar to the human PAB domain (Supplementary Figure S3D). This assignment is particularly notable because budding yeast contains no PARP proteins, and indeed important PARP1-binding residues are not conserved in the fungal proteins (Supplementary Figure S3C). Surprisingly, these residues are conserved in CR-Timeless.

### Interactions of the Tof1–Csm3 complex

**Figure 3.**
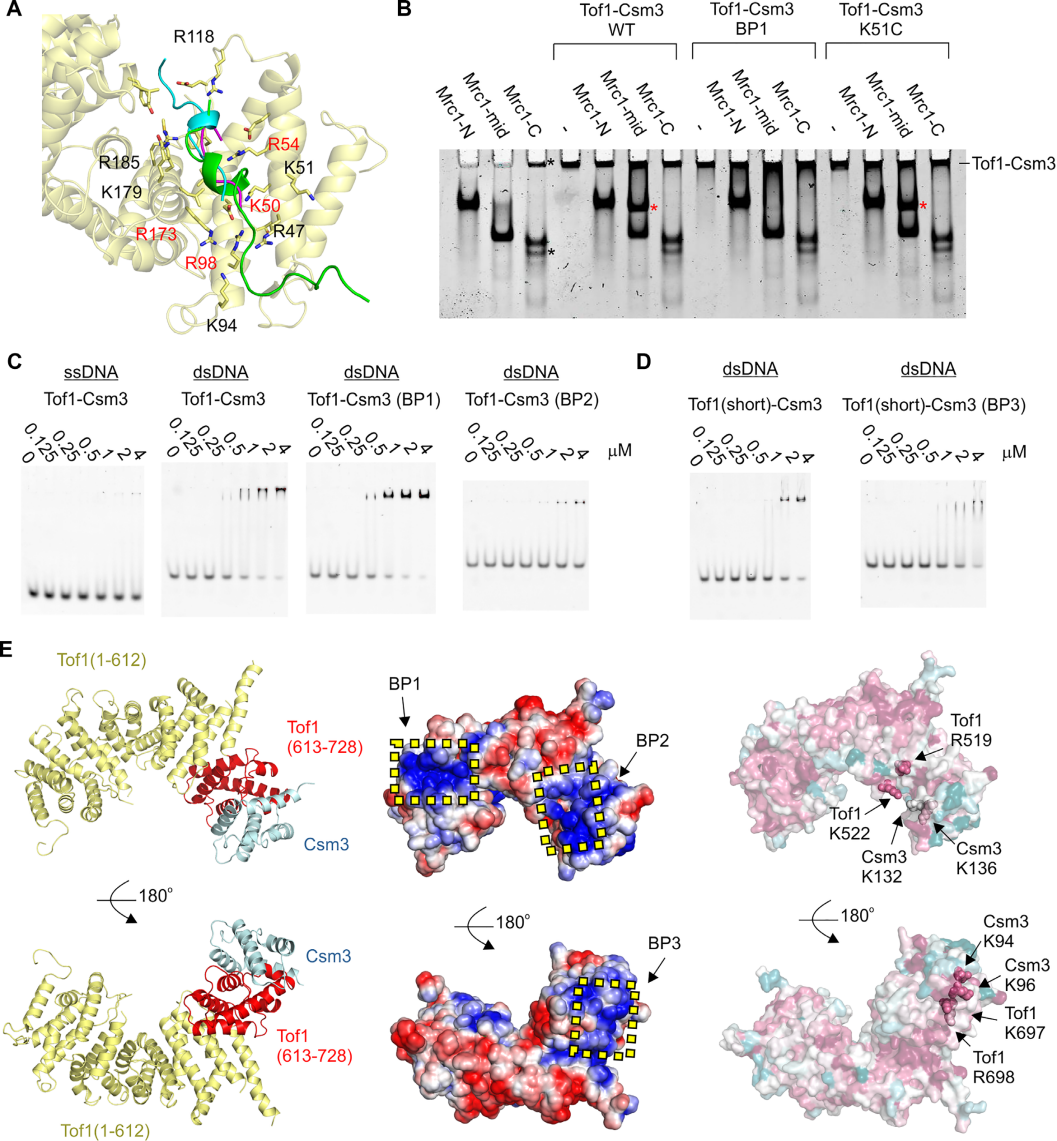
Interactions of the Tof1–Csm3 complex. (**A**) Interaction between Tof1 and a symmetry-related purification tag. The peptide interacting with each copy of Tof1 was superimposed by aligning the interacting elements of Tof1, and is shown in cartoon representation. The N-terminus of Chain E (interacting with Tof1 chain C) is in green, the N-terminus of Chain A (interacting with Chain E) is in cyan, and the N-terminus of Chain D (interacting with Chain A) is magenta. BP1 mutated residues are indicated in red (**B**) Native PAGE analysis of the interaction of Tof1–Csm3 with Mrc1. The indicated proteins were loaded at a concentration of 5 μM. The red asterisks indicate the new band formed when Mrc1-mid and Tof1–Csm3 are mixed. The black asterisks indicate impurities in the Mrc1-C preparation (**C, D**) EMSA gels showing DNA binding properties of the indicated Tof1–Csm3 complexes and variants to 50 nM of either ssDNA or dsDNA. (**E**) Left panel – The portion of Tof1 retained in the minimal complex is shown in red. Middle panel – Surface potential of the Tof1–Csm3 complex. The potential was calculated using APBS (64). Positively charged surface is colored blue and negatively colored red. The regions mutated in BP1, BP2 and BP3 are indicated. Right panel – Surface conservation of the Tof1–Csm3 complex as calculated by the ConSurf sever (65). Magenta indicates high sequence conservation, while cyan indicates low conservation. Residues mutated in BP2 and BP3 are shown in sphere representation and labeled.

Other Armadillo repeat proteins, such as β-catenin and importin-α, often bind peptides within the interior of their α-solenoid structure (55,56) (Supplementary Figure S5). As previously noted (34), there is a highly conserved cleft within this interior towards the N-terminus of Tof1, lined with basic and hydrophobic residues. In our structure, the purification tag of a symmetry copy occupies this cleft (Figure 3A, Supplementary Figure S5). Furthermore, this interaction occurs in all three copies in the asymmetric unit despite the lack of symmetry in the packing, which suggests the cleft has a very high propensity for peptide binding (Figure 3A). Intriguingly, in human Timeless, Arg40 (*C. thermophilum* Lys51), which coordinates a sulfate ion bound in the equivalent cleft (Supplementary Figure S4B) (34), has been found mutated six times to cysteine and once to proline in different cancers in the COSMIC Sanger database (57). This is the most common cancer-associated missense mutation of Timeless, with the second being Pro1043 (five times), which is a key PARP1-interacting residue (Supplementary Figure S3C). A thermal shift assay shows that the equivalent mutation in CtTof1 (K51C) has no effect on the stability of the protein (Supplementary Figure S4A), suggesting that Timeless Arg40 has functional rather than structural importance. Additionally, other residues in this cleft, such as Arg47 and Arg54, are highly conserved in fork protection Tof1/Timeless but not CR-Timeless (Figure 2). To test the role of this cleft, I constructed a quadruple alanine mutation of residues K50/R54/R98/R173, which will be referred to as Basic Patch-mutated 1 (BP1) (Figure 3A).

One well-known interactor of the Tof1–Csm3 complex is the checkpoint mediator and replisome component Mrc1 (Claspin in humans) (11,58). Considering the entirety of Mrc1 is predicted disordered, it could potentially bind like a peptide. To test whether the proteins interact, I generated three maltose binding protein-fused constructs containing the N-terminus (Mrc1-N, 1-400), middle (Mrc1-mid, 401–800) and C-terminus (Mrc1-C, 801–1096) of Mrc1 from *Saccharomyces cerevisiae*. Mrc1-N and Mrc1-mid expressed well, whereas Mrc1-C exhibited some degradation. In a native PAGE experiment, the Tof1(1–900)ΔL1,3–Csm3 construct showed a clear interaction with Mrc1-mid, but not Mrc1-N and Mrc1-C, as seen by a new band forming in between the bands of the individual proteins (Figure 3B, compare lane 6 to lanes 2 and 4). Remarkably, the BP1 mutation completely disrupted the interaction (Figure 3B, lane 10), revealing that Mrc1 is likely to bind to Tof1 in a similar manner to the N-terminal purification tag in my structure. However, the K51C mutation had no effect on the interaction (Figure 3B, lane 14).

Considering the positive charge of the cleft, I tested if it could also function as a DNA binding site. In an EMSA, the Tof1(1–900)ΔL1,3–Csm3 construct bound to dsDNA but not ssDNA with a low micromolar affinity (Figure 3B). However, the BP1 mutation had no effect on this activity (Figure 3B). To map the DNA binding activity of the complex I first investigated whether binding still occurred with a minimal complex of Tof1–Csm3 containing only helices α22–28 of Tof1 (613–728) with Csm3 (Figure 3E, Supplementary Figure S1A). Interestingly, this minimal complex retained dsDNA binding activity (Figure 3C), although *circa* 2-fold weaker, suggesting this region of the complex contains a significant portion of the dsDNA binding site. To further pinpoint the site, I compared the surface potential and sequence conservation to construct two further basic patch mutations: BP2 (Tof1–R519A/Tof1–R522A/Csm3–K132A/Csm3–K136A), and BP3 (Tof1–K697A/Tof1–R698A/Csm3–K94A/Csm3–K96A) (Figure 3E). BP3 was constructed within the minimal Tof1(613–728)–Csm3 complex, while BP2 involved Tof1 residues outside this construct and so was made within Tof1(1–900)ΔL1,3–Csm3. An EMSA analysis showed that, while the BP3 mutation had no obvious effect on dsDNA binding (Figure 3D), the BP2 mutation resulted in a strong dsDNA binding defect (Figure 3C). Although a small amount of shifted complex is seen, the amount of free DNA does not significantly decrease even at the highest protein concentration, unlike for other variants.

## DISCUSSION

The Tof1–Csm3 complex and its human orthologue the Timeless–Tipin complex have been shown to have a number of important functions at the replication fork. By solving the structure of this complex we now can begin to understand what molecular role the complex plays to link these processes together. Importantly, the Tof1–Csm3 and Timeless–Tipin complexes are highly related (Figures 1B and 2), and thus likely play the same role at the fork, while the Drosophila CR-Timeless seems to have the same overall fold but clear differences in sequence related to its separate role (Figure 2) (31), i.e. interaction with the Period (59) and Cryptochrome proteins (60).

Rather than consisting of multiple distinct domains, the core of the Tof1–Csm3 complex forms a single large alpha-helical repeat protein. Thus, some caution should be taken with any previous functional studies which artificially truncated this fold. This structure suggests the protein has a scaffolding or mechanical role at the fork, which is presumably further regulated by the large intrinsically disordered regions in both Tof1 and Csm3. A recent 2D cryo-electron microscopy study has shown that Tof1–Csm3 binds ahead of the replicative helicase and reduces the flexibility of incoming DNA (35). Such a mechanical function would fit well with my structure, given its rigid structure and dsDNA binding properties. My structure also reveals a highly conserved peptide-binding patch and I show this interacts with the middle of Mrc1. Intriguingly one arginine in this binding patch has been detected as a cancer-associated mutation. This mutation in CtTof1 does not significantly affect the Mrc1 interaction, but the molecular details of the human Timeless-Claspin interaction may differ. Normally, Timeless is overexpressed in cancer, and cells become dependent on the protein to combat cancer-caused replication stress (6). Future studies will be able to ascertain whether this mutation has a positive or negative effect on the Timeless-Claspin interaction and whether this affects replisome progression or the S phase checkpoint.

While this work was in revision, a cryo-EM structure of yeast Tof1–Csm3 bound to the replisome was published (61). Their structure of Tof1–Csm3 is highly similar to mine but further shows that it binds to the front of the CMG helicase and interacts with upstream DNA *via* the region affected by my BP2 mutation, as well as flexible parts missing in my structure.

Overall, my structure provides a basis for understanding the interactions, mutations and function of both the fork protection complex and circadian rhythm regulator Timeless protein at a molecular level.

## DATA AVAILABILITY

Coordinates and structure factors for the Tof1–Csm3 complex have been deposited in the protein data bank under the PDB code 6XWX.

## Supplementary Material

gkaa456_Supplemental_File
