# Supplementary material for: Crystal structure and interactions of the Tof1–Csm3 (Timeless–Tipin) fork protection complex
Source: Nucleic Acids Res. 2020 May 29;48(12):6996–7004. doi: 10.1093/nar/gkaa456 (PMC7337906; doi:10.1093/nar/gkaa456)
Supplement: gkaa456_Supplemental_File [file gkaa456_supplemental_file.pdf]

Supplementary Information

Figure S1 – Crystallization strategy. (**A**) Constructs screened for crystallization. 'Short' contains only the indicated region, while 'long' contains the indicated region additional. Tof1-C only contains the 'long' region. 'NaCl' refers to the concentration of sodium chloride required to solubilize the complex. (**B**) Design of homemade crystallization screens for challenging complexes. One screen contains PEGs 4K, 6K and 8K, while the other contains 10K, 12K and 20K. All salt is used at a concentration of 200 mM. The salts (numbered 0-15) and the high and low PEG concentrations are described below the schematic.

Figure S2 – Quality of the electron density and model fit. Models are shown in cartoon and stick representation with the same coloring as Fig. 1. 2Fo-Fc maps are shown for (**A**) The entirety of the Tof1-Csm3 complex (Chain A-Chain B), (**B**) A low B-factor region of Tof1, (**C**) The interface between Tof1 and Csm3, (**D**) The N-terminal purification tag of Chain E (interacting with Tof1 chain C).

Figure S3 – Sequence analysis of Csm3 and parts of the Tof1-Csm3 complex which were not crystallized. Alignments were prepared as in Fig. 2. (**A**) Alignment of the region Csm3/Swi3/Tipin homologues present in my constructs (**B**) Alignment of Loop 1 (Fig. S1A) from Tof1/Timeless homologues, excluding CR-Timeless, as the sequence and size are very different. (**C**) Alignment of the putative PAB domains from Tof1/Timeless homologues. Arabidopsis Timeless was excluded as no PAB domain is predicted to be present. Loop4 is shown in Fig. S1A. Asterisks indicate PARP1-interacting residues in Timeless PAB. (**D**) Comparison of the fold of the Timeless PAB domain (PDB 4XHT) with the structure of the C-terminal sequence of Chaetomium Tof1 generated *de novo* by the RaptorX server (Wang *et al.*, 2017)

Figure S4 – Position of the Timeless cancer mutation and thermal shift analysis of the Tof1-Csm3 constructs and mutants. (**A**) A superposition of Tof1-Csm3 and human Timeless (pdb 5MQI) as in Fig. 1B, with the purification tags bound to Tof1 shown as in Fig. 3A. The cancer mutated residue, R40, has alternative conformations and both are shown in stick representation. (**B-D**) Thermal shift analysis by Thermofluor showing unfolding of the indicated constructs and variants. Representative curves are shown while T_M values are the average of three measurements.

Figure S5 – Examples of peptide binding by Armadillo-repeat proteins. The Armadillo-repeat protein is shown in gray, and the peptide in red. Left panel – β-Catenin/E-Cadherin (Huber and Weis, 2001), middle panel – importin-α/SV40 T antigen nuclear localization signal (Conti *et al.*, 1998). Right panel – one Tof1-Csm3 peptide binding example from Fig. 3A.

A

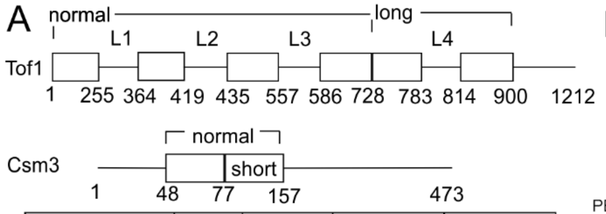

Construct	NaCl (mM)	Crystals	Diffraction	Solved
Tof1-Csm3	500	-	-	-
Tof1-Csm3 DL1/DL3	500	Yes	V. weak	-
Tof1-Csm3 DL1a/DL3	500	No	-	-
Tof1-Csm3short DL1/DL3	500	Yes	No	-
Tof1long-Csm3 DL1/DL3	200	No	-	-
Tof1long-Csm3 DL1/DL3/DL4	200	Yes	No	-
Tof1-Csm3 DL1/DL2/DL3	500	Yes	3.1 A	Yes
Tof1-Csm3short DL1/DL2/DL3	500	Yes	No	-
Tof1-C	0	Yes	2.9 A	No – Severe crystal pathologies
Tof1-C DL4	0	No	-	-
NtruncTof1-Csm3	200	Yes	No	-
NtruncTof1long-Csm3	50	Yes	No	-
NtruncTof1long-Csm3 DL4	50	Yes	No	-

B

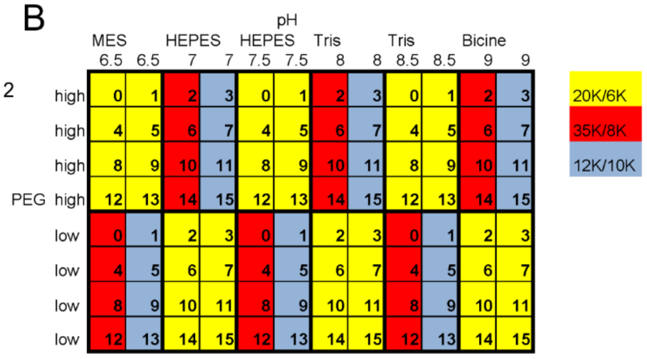

high
- 12% PEG 20K
- 14% PEG 10K/12K
- 15% PEG 4K/6K/8K

low
- 6% PEG 20K
- 7% PEG 10K/12K
- 7% PEG 4K/6K/8K

0. No Salt
1. Magnesium chloride
2. Magnesium sulfate
3. Magnesium acetate
4. Magnesium formate
5. Sodium chloride
6. Potassium chloride
7. Potassium iodide
8. Ammonium sulfate
9. Ammonium chloride
10. Lithium nitrate
11. Lithium sulfate
12. Sodium sulfate
13. Potassium sulfate
14. Potassium formate
15. Potassium acetate

Figure S1

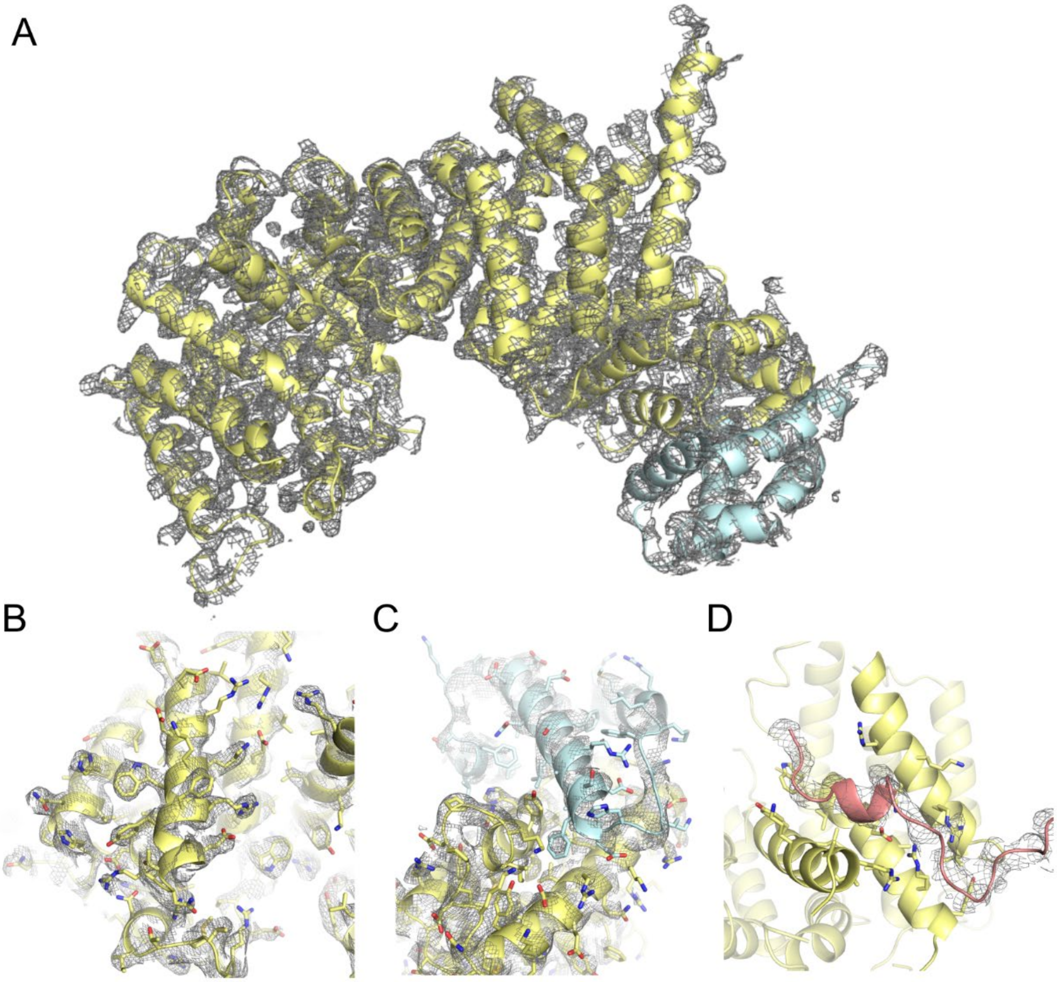

Figure S2

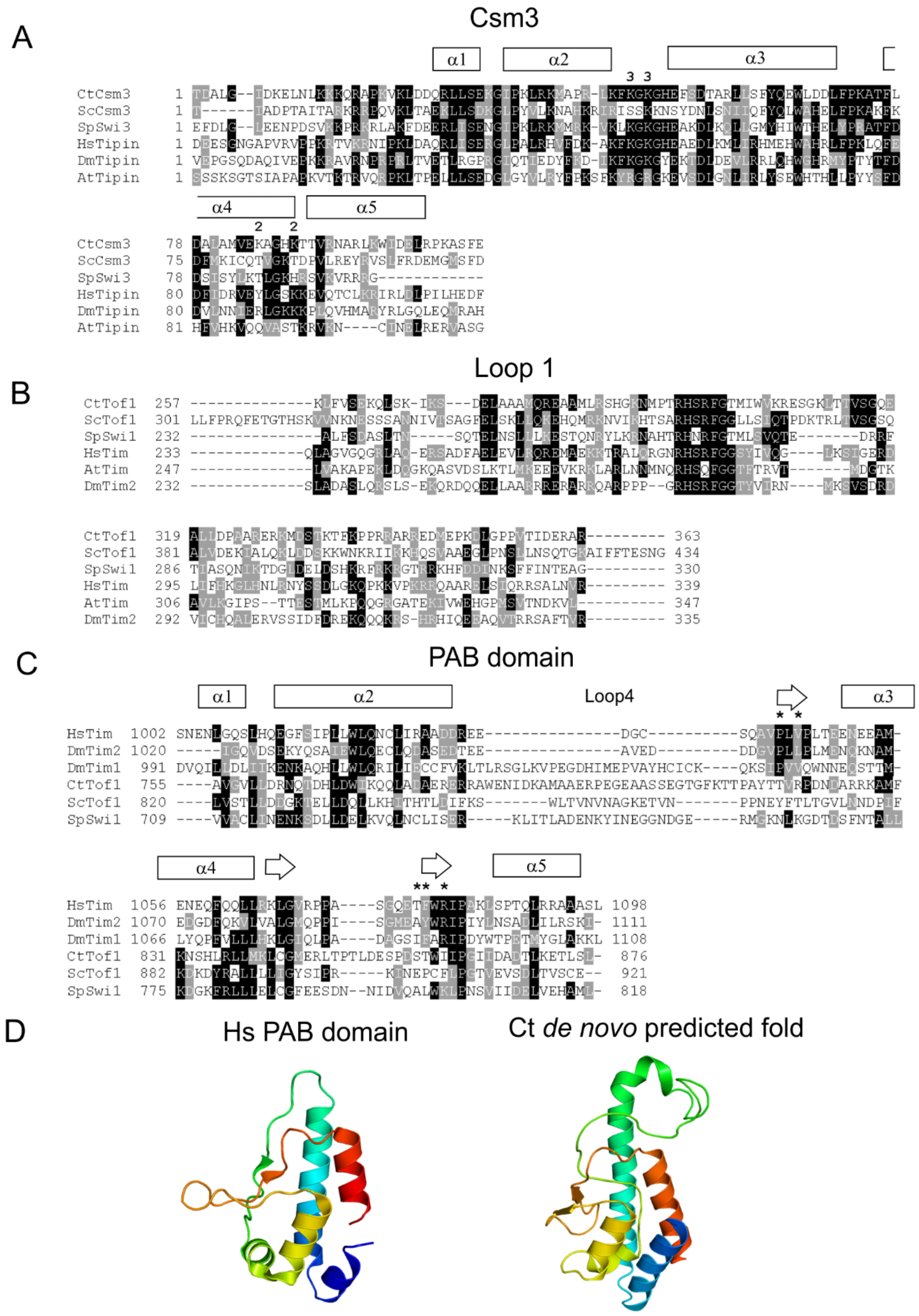
A
Csm3
α1
α2
α3
α4
α5
CtCsm3
ScCsm3
SpSwi3
HsTipin
DmTipin
AtTipin
B
Loop 1
CtTof1
ScTof1
SpSwi1
HsTim
AtTim
DmTim2
C
PAB domain
Loop4
HsTim
DmTim2
DmTim1
CtTof1
ScTof1
SpSwi1
D
Hs PAB domain
Ct de novo predicted fold

Figure S3

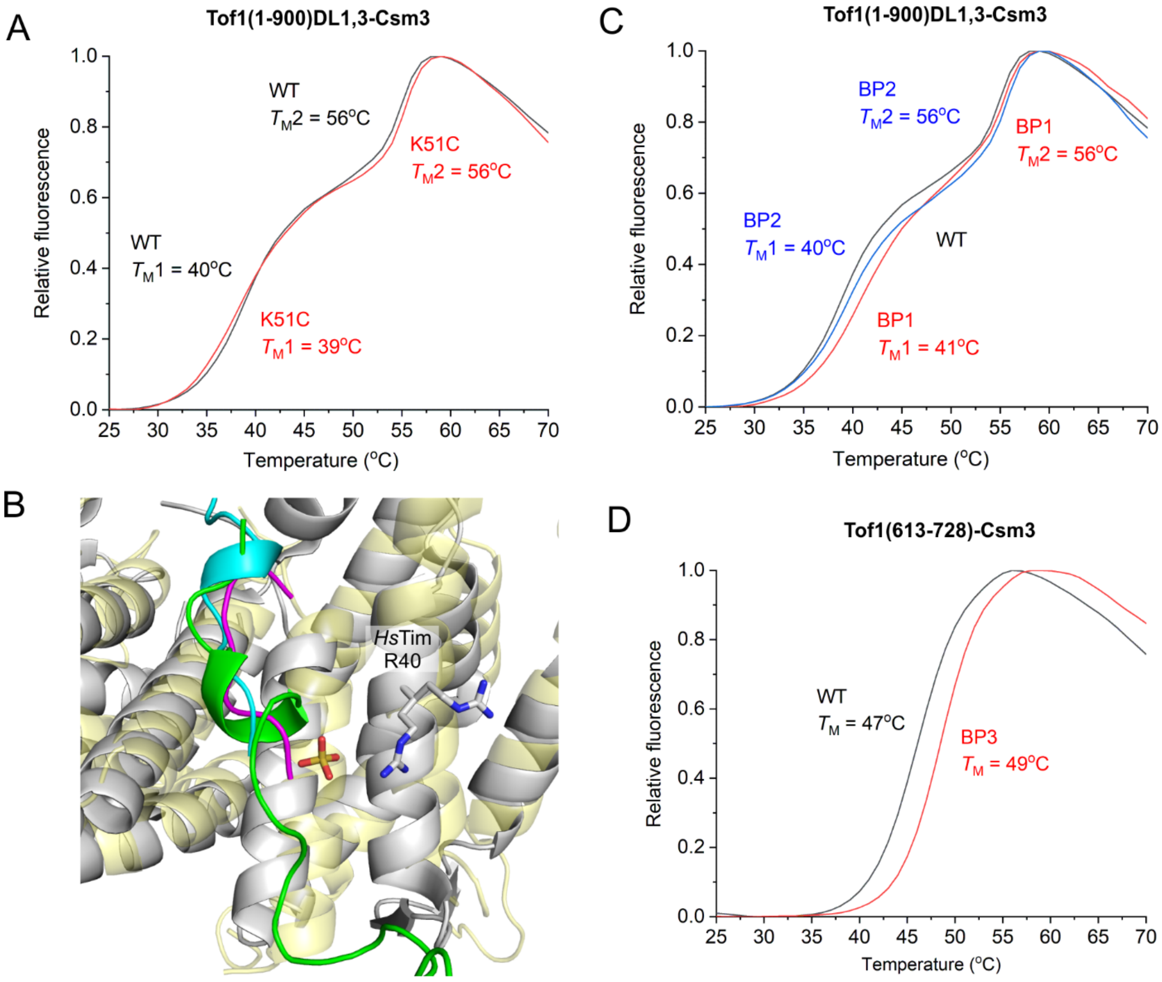
A
Tof1(1-900)DL1,3-Csm3
Relative fluorescence
Temperature (°C)
WT
T_M2 = 56°C
K51C
T_M2 = 56°C
WT
T_M1 = 40°C
K51C
T_M1 = 39°C
B
HsTim
R40
C
Tof1(1-900)DL1,3-Csm3
BP2
T_M2 = 56°C
BP1
T_M2 = 56°C
BP2
T_M1 = 40°C
WT
BP1
T_M1 = 41°C
D
Tof1(613-728)-Csm3
WT
T_M = 47°C
BP3
T_M = 49°C

Figure S4

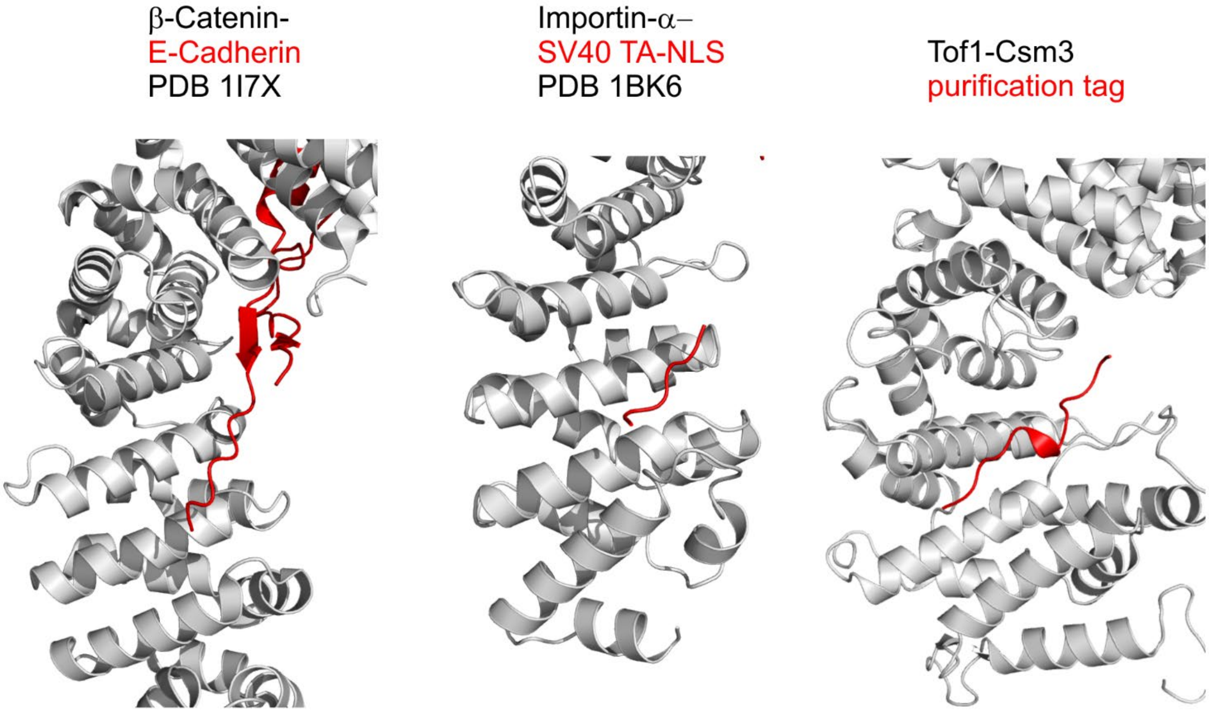

β-Catenin-
E-Cadherin
PDB 1I7X
Importin-α–
SV40 TA-NLS
PDB 1BK6
Tof1-Csm3
purification tag

Figure S5

Table S1 – Primer list

Name	Sequence (5’ -> 3’)
P1	CAAGACCCATGGAAGATGGTGAGGTTAACGATG
P2	CAAGACGGATCCTTATTGTTTCTCAAAGCCGTATTCCAG
P3	CAAGACAAGCTTTTATTCAAATGATGCTTTCGGGCGGAG
P4	CAAGACGAATTCAAGAAGGAGATATACCATGACAGATGCGCTGGGTATCGAC
P5	CAAGACGAATTCAAGAAGGAGATATACCATGTCGGAAAAAGGTATTCCTAAACTTCG
P6	GTCAGCAGCTTGGTAGCTTTGTTTCAG
P7	CTTTAGGGTCCACGCGTTTCACC
P8	GCACGCCAGCAGCTTGGTAGCTTTGTTTCAG
P9	GCGTTCACCTTTAGGGTCCACGCGTTTCAC
P10	TCCAGTTTTAATTTAATTGCCTCCGTGC
P11	ACGGCGCGCGCGTTCGG
P12	GTTCGGCGGACGATGAAAAAATGG
P13	CGGATCGCACCTGAAGGTCTACG
P14	CAAGACGGATCCACTGTTTCATCTAATCCACGTCCG
P15	CAAGACGAATTCTTATTTGCGGCGCAATTGATGTTCAGC
P16	ACTGTTTCATCTAATCCACGTCCG
P17	TTGTTTCTCAAAGCCGTATTCCAG
P18	GTGCATACACCACCGTTCGCCC
P19	CCGCTCGACGTTCGCGTTCCG
P20	ACCCCACAAGGCGTGGTAGATACG
P21	TACATCGTTAACCTCACCATCTTCC
P22	AAAATGGATTCGTTTCTATGACGAAAAAACTAACC
P23	GCCAGGTCCCGAAGGACTTCC
P24	TTTCTATGACGAAAAAACTAACCGCATGG
P25	GCAATCCATTTTGCCAGGTCCCGAAGG
P26	CTATCGCATTGGCGTGCTTCGAACTG
P27	CCGCTTTGTATTTATTCGAGTTTTCATTCG
P28	CCGACCAAGGCATCATAAAACTGATTCTG
P29	CTGCGGTTCGTTCTCCGATCGG
P30	ACCTATGCAGATCAGGGTTTTGAATAC
P31	AGCTACAATATTGGCCACGGCATCG
P32	GCCTGCATTCGTAAATTAGAAGAGCGC
P33	TGCCAGAATCTGGCGCACCAGTTCTTCC
P34	GGTGCAGGCCACGAATTTAGCGACACG
P35	CGCAAATTTAAGGCGCGGAGCCATTTTGC
P36	GCAGCGGGCCATGCAACCACC
P37	CTCAACCATAGCCAACGCATCC
P38	CAAGACCCATGGATGATGCCTTGCATGC
P39	CAAGAAGGATCCTTAGCATTTGCAGGAGTCAATTTCCCG
P40	CAAGAACCATGGCAAAATGCATAACACTGGACCTCGACAG
P41	CAAGAAGGATCCTTAATTTTTTGCTCTTTTATTACGAAATCCTCC
P42	CAAGAACCATGGCAAGCTTAGAGCTAGAACTAAGTGATGATG
P43	CAAGAAGGATCCCTAATTATCAAAGCTATCTTGTCCGC

Supplemental References

Conti, E., Uy, M., Leighton, L., Blobel, G. and Kuriyan, J. (1998) 'Crystallographic analysis of the recognition of a nuclear localization signal by the nuclear import factor karyopherin alpha', *Cell,* 94(2), pp. 193-204.

Huber, A. H. and Weis, W. I. (2001) 'The structure of the beta-catenin/E-cadherin complex and the molecular basis of diverse ligand recognition by beta-catenin', *Cell,* 105(3), pp. 391-402.

Wang, S., Sun, S., Li, Z., Zhang, R. and Xu, J. (2017) 'Accurate De Novo Prediction of Protein Contact Map by Ultra-Deep Learning Model', *PLoS Comput Biol,* 13(1), pp. e1005324.
